# Improving OB/GYN Resident Ultrasound Education Through a Simulation-Based Curriculum

**DOI:** 10.7759/cureus.104115

**Published:** 2026-02-23

**Authors:** Ilina Terziyski, Luke P Hansen, Shena Dillon

**Affiliations:** 1 Obstetrics and Gynecology, Baylor Scott & White All Saints Medical Center, Fort Worth, USA

**Keywords:** gynecology, obstetrics, obstetrics and gynecology residency, obstetrics & gynecology, resident education, simulation, skills and simulation training, sonogram, ultrasongraphy obstetrics, ultrasound (u/s)

## Abstract

Introduction: Ultrasound is a fundamental skill of practicing obstetrician-gynecologists with proficiency ideally obtained in training. However, ultrasound training in residency has not been standardized and varies based on clinical experience and the number of ultrasounds performed. Simulation provides a way to standardize training while incorporating hands-on skills. This study evaluated the effects of a simulation-based ultrasound curriculum on resident confidence and the number of cases logged.

Methods: This is a prospective study comparing the differences in end-of-year postgraduate year 1 (PGY1) obstetric/gynecology (OB/GYN) resident confidence and case logs in ultrasonography with and without a simulation curriculum. PGY1 residents from the 2022-2023 cohort did not train on a simulation curriculum prior to the start of their clinical training, while the PGY1 residents from the 2023-2024 class underwent both curricular training and simulated image capture. Although some residents may have had some didactic exposure to ultrasound in medical school, that experience was not taken into account. Both groups had confidence scores and case logs obtained at the end of their PGY1 year. Paired Student t-tests were performed.

Results: The 2022-2023 cohort performed on average 153 transvaginal and 29 obstetric sonograms. The 2023-2024 cohort performed on average 130 transvaginal and six obstetric sonograms. The end-of-year questionnaire showed no significant difference in ultrasound confidence between the two cohorts (all items p > 0.17).

Conclusions: Although the class that underwent simulation training had fewer ultrasound numbers per resident, they showed confidence ratings similar to the class with more cases. With simulation, fewer clinical sonograms may be needed to achieve the same confidence level. A standardized simulation curriculum could be a helpful adjunct to residency programs to address variance in clinical ultrasound training, and a larger-scale study to confirm these preliminary findings would be justified.

## Introduction

Ultrasound is one of the most basic skills used daily by practicing obstetricians and gynecologists. Its utility is both cost-effective and safe for patients [1]. With its availability at bedside and the increasing resolution of ultrasound imaging, ultrasonography is the first-line modality for the diagnostic workup of most obstetric and gynecologic (OB/GYN) cases [2]. Therefore, proficiency in ultrasound is essential to the role of an OB/GYN. Ideally, this training with sonography should occur during residency. However, ultrasound training for OB/GYN residents has not been standardized [3]. Furthermore, ultrasonography is operator-dependent, and results can vary drastically between sonographers depending on their skill level [4].

Resident training in sonography is usually determined by the number of clinical experiences and ultrasound cases performed. Clinical experiences can vary between residents, even among residents from the same OB/GYN program due to clinical volume fluctuations per rotation. A resident’s confidence in their sonography ability also varies person-to-person despite the number of sonograms they have completed [5]. Sonography skills can be acquired, improved, and assessed using a high-fidelity simulator [6,7]. An obstetrical ultrasound simulator is as good a method as standardized patient examination for evaluating practical skills in trainees following structured training in obstetrical ultrasound [8]. Sonography simulators may even be better than clinical learning when it comes to technical performance like image optimization [9].

The purpose of this study is twofold: (i) To quantify resident confidence in their obstetric and gynecologic ultrasonography skills with and without simulation training and (ii) To compare obstetric and gynecologic ultrasound volumes in the postgraduate year 1 (PGY1) year with and without simulation training. We plan to quantify OB/GYN resident confidence in ultrasound skills and volume of cases before and after implementation of SonoSim(R) curriculum to better evaluate sonogram training in our graduate medical education program. Our primary outcome is resident confidence with ultrasound, and we hypothesize that simulation is non-inferior to real-world ultrasound experience.

## Materials and methods

This study is a two-independent group cohort design comparing the differences in PGY1 end-of-year confidence in ultrasonography with and without the SonoSim(R) curriculum using a validated confidence questionnaire (see the Appendix) [10]. SonoSim(R) is an ultrasound simulation software designed to teach sonography with hands-on training combined with didactic instruction. All 12 residents in the first and second classes at the Baylor Scott & White All Saints OB/GYN residency were included. No residents were excluded, though they could opt out of participation. Because we cannot control for the varying levels of medical school exposure to ultrasound in each new residency class, prior ultrasound exposure was not taken into account. Residents in this study who had ultrasound experience prior to residency had limited didactic exposure to pelvic ultrasound, but none had performed transvaginal ultrasounds or obstetric ultrasounds beyond fetal presentation and placentation. 

**Figure 1:**
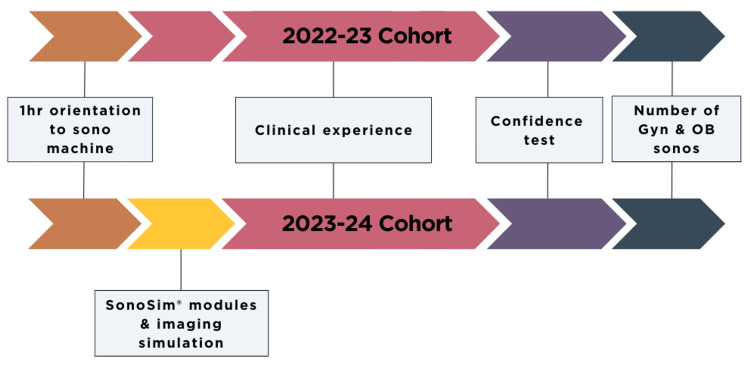
Overview of the Project: 2022-2023 PGY1 residents did not have SonoSim(R) curriculum prior to clinical experience and were assessed with an end-of-year confidence questionnaire as well as case volumes. 2023-2024 PGY1 residents did have standard OB/GYN sonography simulation curriculum (SonoSim(R)) prior to starting clinical experience and were assessed with an end-of-year confidence questionnaire and case volumes.

Residents in the 2022-2023 cohort did not use the SonoSim(R) curriculum prior to clinical experience during their PGY1 year. They completed an end-of-year (June 30th, 2023) sonography confidence assessment, having only had clinical experience to build their skills. Residents in the 2023-2024 cohort used the standardized SonoSim(R) curriculum prior to starting PGY1 year and completed an end-of-year (June 30th, 2024) confidence assessment (Figure 1). All residents in both cohorts had clinical experience performing ultrasound under direct faculty guidance during their PGY1 year, both transvaginal gynecologic ultrasound in the clinic and OB ultrasound at various gestational ages in the obstetric emergency room and in the clinic. The number of ultrasounds per resident was tracked via the ACGME logging database.

2023-2024 residents completed the SonoSim(R) modules pertaining to obstetrics and gynecology within the first 60 days of residency, which include lessons on image acquisition, scanning technique, normal and pathologic sonographic anatomy, and imaging adjuncts, as well as passing a mastery test at the end of each module. Each module has several simulation patient cases to help residents practice their skills using the provided simulation probe. Modules in the curriculum included: (i) Basic Female Pelvis; (ii) Core OB-GYN; (iii) Core Six-Step Obstetric Exam; (iv) OB 1st Trimester Pregnancy; (v) OB 2nd & 3rd Trimester Pregnancy (Parts I & II); (vi) GYN Normal Uterus; (vii) GYN Abnormal Uterus (Parts I & II); (viii) GYN Normal Adnexa; (ix) GYN Nonmalignant Adnexa; (x) GYN Malignant Adnexa

The pre- and post-questionnaire results within the 2023-2024 PGY1 cohort were examined. End-of-year (June 30th, 2023 vs. June 30th, 2024) questionnaire results were compared between the 2022-2023 cohort and the 2023-2024 cohort [10]. Paired Student t-tests were performed with a significance of p <0.05.

## Results

Residents in the 2022-2023 cohort performed 153 transvaginal and 29 obstetric sonograms on average. Residents in the 2023-2024 cohort performed 130 transvaginal and six obstetric sonograms on average. The end-of-year (June 30th, 2023 vs. June 30th, 2024) questionnaires showed no significant difference in ultrasound confidence between the two cohorts (Table 1).

**Table 1:** End-of-year confidence scores in the 2022-2023 cohort vs. 2023-2024 cohort

Questionnaire item	Class without SonoSim(R) (n = 6)	Class with SonoSim(R) (n = 6)	p-value	t-value
I am certain that my sonography skills are correct.	3	3.5	0.17	1.58
I feel that I can perform a sonogram without hesitation.	3.67	3.67	1	0
My sonography skills would convince an observer that I’m competent.	3.17	3.5	0.48	0.75
I feel sure of myself as I perform a sonogram.	3.17	3.33	0.66	0.46
I feel satisfied with my ability to perform a sonogram.	3.33	3.33	1	0

There was a significant increase from beginning-of-year to end-of-year confidence within the 2023-2024 cohort (Table 2).

**Table 2:** Beginning vs. end of year confidence in the 2023-2024 Cohort (SonoSim(R) Cohort)

Questionnaire item	Beginning of Year (n = 6)	End of Year (n = 6)	p-value	t-value
I am certain that my sonography skills are correct.	2	3.5	0.017	3.5
I feel that I can perform a sonogram without hesitation.	1.67	3.67	0.012	3.87
My sonography skills would convince an observer that I’m competent.	1.67	3.5	0.012	3.84
I feel sure of myself as I perform a sonogram.	1.83	3.33	0.017	3.5
I feel satisfied with my ability to perform a sonogram.	1.67	3.33	0.004	5

## Discussion

This study found no significant difference in confidence between residents trained with or without a simulation curriculum. However, the group with simulation experience had fewer clinical cases with no observed decrease in confidence. These findings suggest that simulation experience could provide similar confidence despite less clinical experience. A larger sample size of an established program that has a set way of teaching sonography before using a standardized simulation curriculum would be more likely to show further impacts of using Sonosim(R) and enhance generalizability.

Our study aligns with what was found by Tolsgaard in that simulation-based training can decrease the need for supervision over time [9]. A resident who feels more confident in their skills also improves the patient’s comfort and perceived confidence in their ultrasound operator [9]. We were particularly interested in ultrasound simulation as a new program, as there would likely be a variety of clinical experiences gained by residents and between classes. Three-quarters of United States OB/GYN residency programs have an obstetric ultrasound rotation in PGY1 year; however, our program differs in that ultrasound experience in PGY1 year is primarily gynecologic and transvaginal [3]. SonoSim(R) allowed residents to supplement their learning PGY1 year and fill any gaps they felt they had clinically. Although not a replacement for real-time feedback from an experienced supervising physician, a self-guided simulation curriculum prior to clinical exposure can enhance resident understanding during real-life clinical scenarios. Simulation can work as preparation to enhance future clinical learning, not only as additional content [9]. Therefore, residents were better prepared to start clinical ultrasound due to the simulation curriculum. Further directions of this study could break down confidence in gynecologic vs. obstetric ultrasound skills, assess ultrasound efficiency in the clinical setting, and assess skill mastery with a standardized simulation.

Limitations of this study include a small sample size, a single center, and a lack of cohort randomization. This study was conducted at a developing residency program with only 12 residents, which could limit its generalizability. Additionally, we noticed that there was a difference in the clinical exposure to obstetrics ultrasounds between our 2022-2023 and 2023-2024 cohorts, likely because during the 2023-2024 academic year, obstetric ultrasounds were being split amongst 12 residents instead of 6, and the fact that the primary clinical exposure to transabdominal obstetric ultrasounds occurs during the second year of residency at our program.

Another limitation of the study is the fact that a subjective survey on personal confidence level was conducted here. A more objective way to measure resident confidence and skill would be to have a standardized exam with a numerical score given to compare competency. A study by Chalouhi et al. found that although simulated ultrasound is not as challenging as ultrasound performed on a live subject, it can be used to evaluate sonographer skill just as effectively and in a uniform way [8].

Having a set simulation curriculum could help residency programs struggling with sonogram experience and skill. A program like SonoSim(R) could increase resident confidence if certain sonogram experience is lacking or if tailored learning is needed to help individuals struggling with a particular sonogram view or case experience. For example, resident training with simulation has been shown to improve procedural performance among radiology residents [11]. Although a didactic ultrasound curriculum has been developed for US residency programs in OB/GYN, a uniform simulation-based curriculum to practice skills would be beneficial as ultrasonography is a kinesthetic skill [6]. Virtual reality simulation has been shown to help novices achieve near-expert-level skills and improve their efficiency once in the clinical setting [12]. SonoSim(R) could be a more budget-friendly way to achieve similar results.

The cost of sonography is often a limiting factor for hospitals and clinical sites. Having a curriculum could potentially save graduate medical education sites money on this vital skill. A study by McLean et al. showed how a low-cost ultrasound simulator improved trauma simulation [13]. A further cost analysis would need to be conducted to better compare costs and see if a simulation program is a more cost-effective way of learning.

## Conclusions

Although the class that had SonoSim(R) had fewer ultrasound numbers per resident, they showed similar confidence ratings to the class that did not have SonoSim(R). With a simulation curriculum, fewer clinical sonograms may be needed to achieve the same confidence level. Simulation could be a helpful adjunct to programs that have fewer numbers of sonograms early in residency.

Our preliminary findings justify the need for a larger-scale randomized study to determine the effects of ultrasound simulation curriculum on OB/GYN resident sonography confidence and skill.

## Figures and Tables

**Figure 2:**
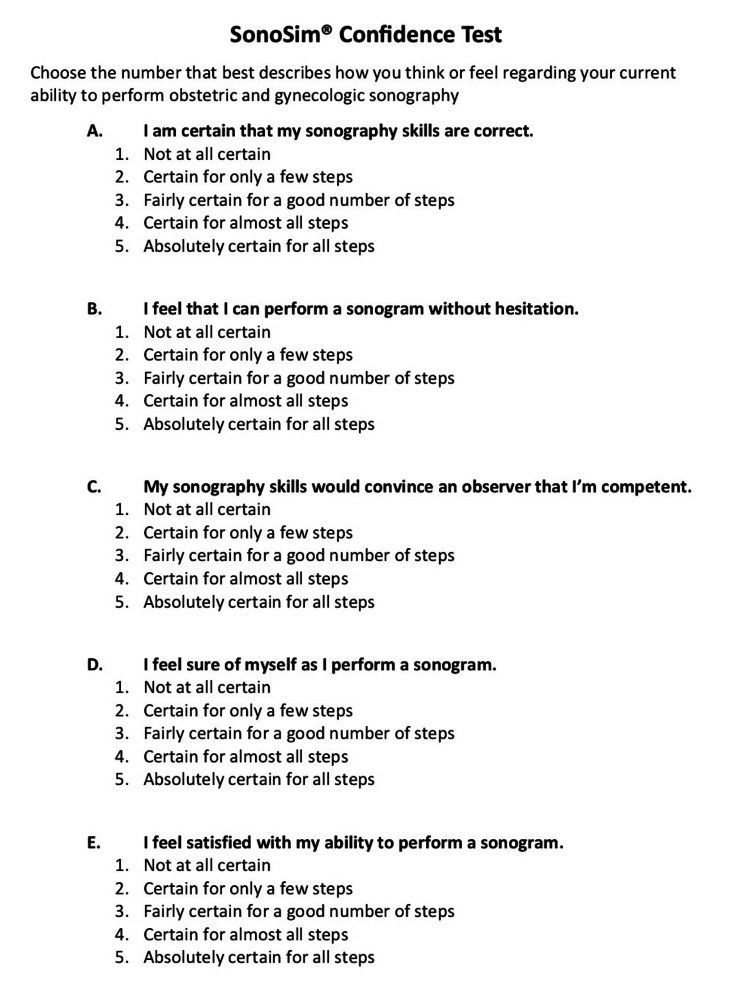
Ultrasound confidence questionnaire Used with permission from [10]
